# Plasmacytoma Variant Translocation 1 (PVT1) Gene as a Potential Novel Target for the Treatment of Diabetic Nephropathy

**DOI:** 10.3390/biomedicines10112711

**Published:** 2022-10-26

**Authors:** Helen Mok, Ahmed Al-Jumaily, Jun Lu

**Affiliations:** 1 School of Science, Faculty of Health and Environmental Sciences, Auckland University of Technology, Auckland 1142, New Zealand; 2 School of Engineering, Computer and Mathematical Sciences, Faculty of Design and Creative Technologies, Auckland University of Technology, Auckland 1142, New Zealand; 3 Maurice Wilkins Centre for Molecular Discovery, Auckland 1142, New Zealand; 4 College of Food Science and Technology, Nanchang University, Nanchang 330031, China; 5 College of Food Engineering and Nutrition Sciences, Shaanxi Normal University, Xi’an 710119, China; 6 School of Perfume and Aroma Technology, Shanghai Institute of Technology, Shanghai 201418, China

**Keywords:** diabetic nephropathy, albuminuria, extracellular matrix accumulation, plasmacytoma variant translocation 1 (PVT1)

## Abstract

**Research in context:**

***What is already known about this subject***:

- The noncoding gene PVT1 was highly expressed in mice and patients with DN;
- PVT1 promotes DN through increasing extracellular matrix (ECM) accumulation in vitro;
- Silencing of PVT1 attenuated secretion of ECM proteins and delayed podocyte apoptosis in vitro.

***What is the key question***:

- Does the inhibition of the noncoding gene PVT1 in a diabetic nephropathy mouse model either prevent or delay the onset of diabetic nephropathy?

***What are the new findings***:

- Silencing of PVT1 ameliorates DN in terms of kidney function in a diabetic nephropathy mouse model independent of blood glucose change;
- The mechanism involves downregulating TGF-β1 and PAI-1 while preserving BMP7;
- PVT1 plays an important role in ECM accumulation and is a potential target for the treatment of DN.

***How might this impact clinical practice in the foreseeable future***:

- The present study demonstrates the application of RNAi-based therapy to delay DN progression in a murine DN model. RNAi-based therapeutics are emerging treatment strategies in cancers, infectious diseases and single gene disorders. There has already been a clinical trial using siRNA for acute kidney injury, resulting in favorable safety data. Our study provided a foundation for a possible clinical long-term RNAi-based prevention/treatment of DN.

**Abstract:**

***Introduction***: Diabetic nephropathy (DN), a severe microvascular complication in patients with diabetes, is clinically characterized by progressive decline in glomerular filtration rate (GFR). DN is the most common cause of end-stage renal disease (ESRD), and has a consistently high mortality rate. Despite the fact that the prevalence of DN is increasing worldwide, the molecular mechanism underlying the pathogenesis of DN is not fully understood. Previous studies indicated PVT1 as a key determinant of ESRD as well as a mediator of extracellular matrix (ECM) accumulation in vitro. More investigations into the role of PVT1 in DN development are needed. ***Objectives***: To study the effect of PVT1 silencing on progression of DN in diabetic male C57BL/6 mice at early, intermediate and relatively advanced ages. ***Methods***: Diabetic mice were treated with either scramble-siRNA (DM + siRNA (scramble)) or PVT1-siRNA (DM + siRNA (PVT1)), whereas the control mice were normal mice without siRNA injection (Control). Blood, urine and kidney were collected at the age of 9 (young), 16 (middle-aged) or 24 (old) weeks old. Kidney function, histology and molecular gene expression were evaluated. ***Results***: Our findings showed that silencing of PVT1 reduced kidney hypertrophy, proteinuria (UAE, UACR, UPE, UPCR), serum creatinine, serum TGF-β1, serum insulin decline, glomerular and mesangial areas, and increased creatinine clearance in diabetic mice to levels closer to the age-matched controls. Also, silencing of PVT1 markedly suppressed the upregulation of PAI-1, TGF-β1, FN1, COL4A1, and downregulation of BMP7. ***Conclusion***: Silencing of PVT1 ameliorates DN in terms of kidney function and histology in diabetic mice. The renoprotection is attributed to the reduction in ECM accumulation, TGF-β1 elevation and insulin decline. PVT1 is suggested to play an important role in ECM accumulation which makes it a possible target for the treatment of DN.

## 1. Introduction

Diabetic nephropathy (DN) is one of the major microvascular complications of diabetes [1]. It is clinically characterized by progressive decline in glomerular filtration rate [2]. It is the most common cause of end-stage renal disease (ESRD) [3]. ESRD resulting from diabetes has been estimated to be 30–47% of all incident cases worldwide [4]. It accounts for over 45% of patients receiving renal replacement therapy (RRT) such as haemodialysis, peritoneal dialysis or kidney transplantation [5]. The global number of patients receiving RRT is projected to reach 5.4 million by 2030 [6], given that rates of ESRD are increasing approximately 8% annually [7], resulting in an enormous healthcare expenditure [8]. Patients with DN and ESRD are associated with poor quality of life and high mortality rates [9]. Therefore, prevention and treatment of DN are crucial to the improvement of health outcomes of diabetic patients and to the reduction of the socioeconomic burden of DN.

The pathogenesis of DN is multifactorial, while genetic susceptibility plays an important part [10]. A landmark genome-wide association study (GWAS) carried out among African Americans with type 2 diabetes provided the first evidence supporting PVT1 as a candidate gene for ESRD [11]. Another GWAS showed PVT1 was associated with ESRD in Caucasians with type 1 diabetes in an ethnically distinct population [12]. Taken together, PVT1 appears to be an important determinant of ESRD across populations with different ethnicities and types of diabetes.

PVT1 is a long noncoding RNA (lncRNA) (1.9 kb) that does not encode proteins [13]. LncRNAs (more than 200 nucleotides in length) regulate transcriptional processes and functions through both activation and inhibition of genes [14]. Although the oncogenic functions of PVT1 are widely reported in a range of cancers [15], its roles in other diseases are poorly understood. PVT1 was highly expressed in mice and patients with DN [16,17]. Recent studies have suggested that PVT1 promotes DN by increasing extracellular matrix (ECM) accumulation [18,19], accelerating podocyte injury and apoptosis [16], and promoting epithelial-mesenchymal transition (EMT) preceding renal fibrosis [20]. Silencing of PVT1 attenuated the secretion of ECM proteins in cultured mesangial cells and delayed podocyte apoptosis [16,18]. However, there are very few studies reported to date elucidating the relationship between PVT1 and DN, particularly in vivo.

This study hypothesizes that PVT1 affects DN development through ECM accumulation. We investigated the effect of silencing PVT1 on progression of DN in a murine model and on its potential mechanism of action. Our findings might contribute to expanding the current knowledge and paving the way for alternative treatment options for DN such as RNAi-based therapy.

## 2. Materials and Methods

### 2.1. Animals

Male C57BL/6 mice were provided by the Vernon Jansen Unit (VJU) of the University of Auckland. They were housed in the animal facility maintained on constant temperature (22–25 °C), relative humidity (55–65%) and 12 h light/12 h dark cycles. After weaning, the mice were fed with either a high fat diet (HFD, 60% kcal from fat; TD06414, Enivgo, Madison, WI, USA) or a low-fat diet (LFD, 10% kcal from fat; TD08806, Enivgo, Madison, WI, USA), and were supplied with tap water *ad libitum*. At 5 weeks of age, the HFD-fed mice were intraperitoneally administered with low dose streptozotocin (STZ; 40 mg/kg of body weight per day; Sigma, St. Louise, MO, USA) for three consecutive days after 4 h fasting, while the LFD-fed mice were administered with vehicle (sodium citrate buffer). Diabetes was induced by the combination treatment of HFD and STZ. The diabetic group and normal control group were represented by the HFD-STZ-treated mice and the LFD-vehicle-treated mice, respectively. The effects of PVT1 silencing on diabetic mice and control mice were investigated using in vivo RNA interference. The diabetic mice were divided into two groups, which were intravenously injected with scramble-siRNA (DM + siRNA (scramble) or PVT1-siRNA (DM + siRNA (PVT1)) according to the manufacturer’s procedural instructions. The proprietary pre-designed PVT1-siRNA, scramble-siRNA, Invivofectamine 3.0 reagent, phosphate buffered saline (PBS) and DNase/RNase-free distilled water were purchased from ThermoFisher Scientific (Carlsbad, CA, USA). The mice were culled at the age of 9 (young), 16 (middle-aged) or 24 (old) weeks old, which represented early, intermediate and relatively advanced stages of diabetes.

### 2.2. Ethics Statement

All animal experiments were performed in accordance with the ethics policies and procedures approved by the animal ethics committee of the University of Auckland, New Zealand (AEC001644).

### 2.3. Blood Glucose, Serum Creatinine, Insulin and TGF-β1 Concentrations

The blood glucose was measured by a glucometer (CareSens, Seoul, Korea). To avoid diurnal variation in blood glucose, all measurements were conducted in the same time slot every week. The blood samples were left to stand for 30 min at room temperature, followed by centrifugation at 2000 rpm for 10 min at 4 °C. Serums were collected and stored at −80 °C until analysis. Serum creatinine concentration was determined by Mouse Creatinine Assay (Crystal Chem, Elk Grove Village, IL, USA) as per the manufacturer’s instructions. Serum insulin and TGF-β1 concentrations were determined by Mouse Insulin ELISA and Mouse TGF-β1 ELISA (Crystal Chem), respectively.

### 2.4. Urine Albumin, Protein and Creatinine Concentrations

Mice were individually housed in the metabolic cages (Tecniplast, Lane Cove, NSW, Australia) for 24 h. The total volume of urine was recorded. The urine samples were centrifuged at 1500 rpm for 10 min at 4 °C. The supernatant was collected and stored at −80 °C until analysis. Urine albumin concentration was determined by a sandwich ELISA (Mouse Albumin ELISA Kit; Bethyl Laboratories, Montgomery, TX, USA). Bradford assay was used to measure urine protein concentration (Bio-Rad, Hercules, CA, USA). Urine creatinine concentration was determined by Jaffe’s reaction (Creatinine Assay Kit (Colorimetric), Abcam, Cambridge, UK) as per the manufacturer’s instructions.

### 2.5. Calculation of Urine Albumin, Protein and Creatinine Clearance

Urine Albumin to Creatinine Ratio (UACR) is a ratio of urine albumin to urine creatinine, which was determined by Urine Albumin (μg/μL)/Urine Creatinine (μg/μL). Urine Albumin Excretion (UAE) is the excretion of albumin in urine over 24 h, which was determined by Urine Albumin (μg/μL) × Volume of 24-h urine (μL). Urine Protein to Creatinine Ratio (UPCR) was determined by Urine Protein (μg/μL)/Urine Creatinine (μg/μL). Urine Protein Excretion (UPE) is the excretion of protein in urine in 24 h, which was determined by Urine Protein (mg/μL) × Volume of 24 h urine (μL). Creatinine clearance is an estimated measure of glomerular filtration rate (GFR), which was determined by Urine Creatinine (μg/μL) × Volume of 24 h urine (ml)/(Serum Creatinine (μg/μL) × 1440 (min)) and expressed as (ml/min).

### 2.6. Kidney Histological Analysis

The frozen sections of non-perfused kidneys were stained with periodic acid Schiff (PAS) followed by Mayer’s hematoxylin (Leica, Wetzlar, Germany), which was one of the standard methods to analyze the kidney tissues. The microscopic images were captured using a Leica ICC50 HD (Leica, Wetzlar, Germany). The glomerular area and mesangial area were semi-quantified by the Image-J software (v.1.53u, National Institutes of Health, Bethesda, MD, USA).

### 2.7. Isolation of Glomeruli

The kidneys were perfused with Dynabeads M-450 Epoxy (Invitrogen, Waltham, MA, USA) as per the manufacturer’s instructions. They were immerged in the RNAprotect Tissue Reagent and kept on ice prior to subsequent isolation of glomeruli. Briefly, the kidney cortex was harvested, digested with collagenase and deoxyribonuclease, selectively filtered by cell strainers, and subject to magnetic attraction for isolation of the glomeruli containing Dynabeads. Collagenase A (Roche, Basel, Switzerland), 100 U/mL deoxyribonuclease I (Invitrogen), 100 μm cell strainer (Merck, Kenilworth, NJ, USA), and magnetic particle concentrator (Invitrogen) were used.

### 2.8. Gene Expression Analysis

Total RNA from the isolated glomeruli was extracted using RNeasy Protect Mini Kit (QIAGEN, Hilden, Germany). The amount of RNA was quantified by Qubit RNA BR Assay Kits (Invitrogen) with the Qubit Fluorometer 2.0 (Invitrogen). The cDNA was synthesized from RNA using SuperScript IV VILO Master Mix (Invitrogen). The reaction mixture containing cDNA, forward and reverse primers of targeted sequences (Invitrogen) and PowerUp SYBR Green Master Mix (Invitrogen) were used for running the real-time PCR on LightCycler 480 (Roche). Primers used were as follows: GADPH forward 5′-GGCAAATTCAACGGCACAGT-3′ and reverse 5′-GTCTCGCTCCTGGAAGATGG-3′; PVT1 forward 5′-AGCGTTGACTTAAGAGATGCCA-3′ and reverse 5′-GATTGCCTCCGGCATGAAGA-3′; TGF-β1 forward 5′-GGACTCTCCACCTGCAAGAC-3′ and reverse 5′-CTGGCGAGCCTTAGTTTGGA-3′; PAI-1 forward 5′-CCGATGGGCTCGAGTATGAC-3′ and reverse 5′-TTCTCAAAGGGTGCAGCGAT-3′; FN1 forward 5′-ATACCGTTGTCCCAGAGGTG-3′ and reverse 5′-GTGGAAGAGTTTAGCGGGGT-3′; COL4A1 forward 5′-GGGAAATCCTGGTGACAGGG-3′ and reverse 5′-AAGGAATGGCCGTGGTTTGA-3′; BMP-7 forward 5′-GTCTGCCAGGAAAGTGTCCA-3′ and reverse 5′-CGAGGCTTGCGATTACTCCT-3′. The relative quantification of real-time PCR data was determined by the comparative threshold cycle method (delta-delta Ct method).

### 2.9. Statistical Analyses

All statistical analyses were performed using the software Prism (GraphPad, San Diego, CA, USA). Data were presented as means ± SEM. The significance of the difference between two groups was evaluated using Student’s *t*-test. One-way analysis of variance (ANOVA) with a Tukey’s Multiple Comparison pos*t*-test was used to evaluate differences among groups. A *p*-value < 0.05 was considered statistically significant.

## 3. Results

### 3.1. Physical and Biochemical Characteristics of Murine DN Models

Mice tend to have higher blood glucose concentrations than humans; a non-fasting blood glucose concentration over 13.8 mmol/L, or preferably a chronic elevation over 16.7 mmol/L is appropriate to consider a mouse diabetic [21]. The HFD-STZ-treated mice became diabetic with steady hyperglycaemia from week 7 onwards (data not shown). Blood glucose of diabetic mice was significantly higher than those of control mice in all young (2.37-fold, *p* < 0.001), middle-aged (2.28-fold, *p* < 0.001) and old (2.34-fold, *p* < 0.001) groups (Table 1). Compared with the age-matched controls, the body weight (young and middle-aged, *p* < 0.05), body weight gain (young and middle-aged, *p* < 0.05), kidney weight (middle-aged and old, *p* < 0.05), and kidney to body weight ratio (middle-aged, *p* < 0.05) were higher in diabetic mice (Table 1). Renal risk markers UAE (2.06-fold, 3.00-fold, 3.29-fold vs. control at young, middle-aged, and old, respectively) and UACR (1.53-fold, 2.08-fold, 2.49-fold vs. control at young, middle-aged, and old, respectively) were significantly increased (Table 1). While remaining unchanged in controls, both UAE and UACR were markedly increased in diabetic mice with advancing age. Serum creatinine, a clinical marker to estimate GFR, was significantly increased in diabetic mice in all young (by 53%, *p* < 0.05), middle-aged (by 63%, *p* < 0.05) and old (by 64%, *p* < 0.05) groups. Creatinine clearance was significantly reduced in diabetic mice of middle-aged (by 38%, *p* < 0.05) and old (by 46%, *p* < 0.05) when compared with the age-matched controls, and it was significantly different between young and old diabetic mice (*p* < 0.05) (Table 1).

### 3.2. Expression of PVT1 and ECM Components in Murine DN Models

**Figure 1:**
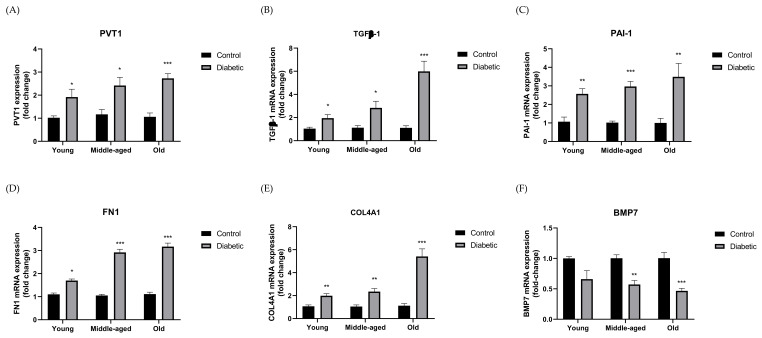
Gene expressions of (**A**) PVT1, (**B**) TGF-β1, (**C**) PAI-1, (**D**) FN1, (**E**) COL4A1, and (**F**) BMP7 of diabetic and control mice of different age groups. The diabetic group and normal control group were represented by the HFD-STZ-treated mice and the LFD-vehicle-treated mice, respectively. Data were presented as means ± SEM, n = 6. A *p*-value < 0.05 was considered statistically significant using unpaired Student’s *t*-test (* *p* < 0.05, ** *p* < 0.01, *** *p* < 0.001 vs. age-matched control). PVT1 (Plasmacytoma variant translocation 1); TGF-β1 (Transforming growth factor beta 1); PAI-1 (Plasminogen activator inhibitor-1); FN1 (Fibronectin 1); COL4A1 (Collagen type IV alpha 1); BMP7 (Bone morphogenetic protein 7).

Expression of PVT1 (Figure 1A), TGF-β1 (Figure 1B), PAI-1 (Figure 1C), FN1 (Figure 1D), and COL4A1 (Figure 1E) was significantly upregulated in HFD-STZ-treated mice when compared with the age-matched controls (*p* < 0.05, *p* < 0.01, *p* < 0.001). In contrast, expression of BMP7 (Figure 1F) was significantly reduced in middle-aged and old diabetic group when compared with the age-matched controls (*p* < 0.01, *p* < 0.001).

### 3.3. Effect of Silencing PVT1 on Blood Glucose, Serum Insulin and TGF-β1

Despite the fact that diabetic mice with PVT1-siRNA had slightly lower blood glucose than did those with scramble-siRNA, both groups had significantly higher blood glucose levels compared with the age-matched controls (Table 2). Compared with the age-matched controls, serum insulin was markedly decreased in diabetic mice with scramble-siRNA at all age groups (*p* < 0.01) and in diabetic mice with PVT1-siRNA at middle-aged and old groups (*p* < 0.05) (Table 2). Serum TGF-β1 was significantly increased in diabetic mice with scramble-siRNA at all age groups when compared with the age-matched controls (*p* < 0.05, *p* < 0.01) and the age-matched diabetic mice with PVT1-siRNA (*p* < 0.05). Old diabetic mice had significantly higher serum TGF-β1 than their young ones with either scramble-siRNA or PVT1-siRNA (*p* < 0.05) (Table 2).

### 3.4. Effect of Silencing PVT1 on Kidney Hypertrophy and Renal Function

Kidney hypertrophy is commonly seen in the early stage of type 1 diabetes in humans and STZ-induced diabetic model [22]. Kidney weight is measured as an index of renal hypertrophy [23]. The kidney to body weight ratio of diabetic mice with PVT1-siRNA were markedly lower than those with scramble-siRNA at middle-aged and old, while the kidney weight was slightly lower (Table 2). UAE was slightly increased from young to old in control group. In contrast, the UAE was significantly elevated as diabetic mice aged. The UAE was significantly higher in diabetic mice with scramble-siRNA of young (by 2.20-fold, *p* < 0.001), middle-aged (by 2.97-fold, *p* < 0.001) and old (by 3.05-fold, *p* < 0.001) groups when compared with the age-matched controls, while a moderate increase in UAE was observed in diabetic mice with PVT1-siRNA (Table 2). When compared with the diabetic mice with scramble-siRNA, the UAE was significantly lowered in diabetic mice with PVT1-siRNA in young (by 37%, *p* < 0.001), middle-aged (by 43%, *p* < 0.01) and old (by 44%, *p* < 0.01) groups. Likewise, diabetic mice with scramble-siRNA had the highest UACR within all age-based groups. The UACR was significantly lowered in diabetic mice with PVT1-siRNA in the young (by 49%, *p* < 0.01), middle-aged (by 32%, *p* <0.05) and old (by 43%, *p* < 0.01) groups when compared with the diabetic mice with scramble-siRNA (Table 2). Compared with the age-matched controls, serum creatinine was significantly increased in diabetic mice with scramble-siRNA in young (by 52%, *p* < 0.001), middle-aged (by 88%, *p* < 0.001) and old (by 73%, *p* < 0.01) groups, while a moderate increase was observed in diabetic mice with PVT1-siRNA. The serum creatinine was significantly lowered in diabetic mice with PVT1-siRNA in young (by 18%, *p* < 0.05), middle-aged (by 25%, *p* < 0.05) and old (by 26%, *p* < 0.05) groups when compared with the diabetic mice with scramble-siRNA. Creatinine clearance decreased gradually in both diabetic groups, while it remained steady in control mice as they aged. The creatinine clearance was significantly declined in diabetic mice with scramble-siRNA of middle-aged (by 41%, *p* < 0.05) and old (by 50%, *p* < 0.01) groups when compared with the age-matched controls, while a smaller decrease (by 12–20%, non-significant) was observed in diabetic mice with PVT1-siRNA (Table 2). When compared with diabetic mice with scramble-siRNA, the creatinine clearance was improved by 50% at middle-age and 60% at old age of diabetic mice with PVT1-siRNA (*p* < 0.05). Taken together, UAE, UACR, UPE, UPCR, creatinine clearance and serum creatinine were not statistically different between diabetic mice with PVT1-siRNA and the age-matched controls (Table 2).

### 3.5. Effect of Silencing PVT1 on Glomerular and Mesangial Areas

**Figure 2:**
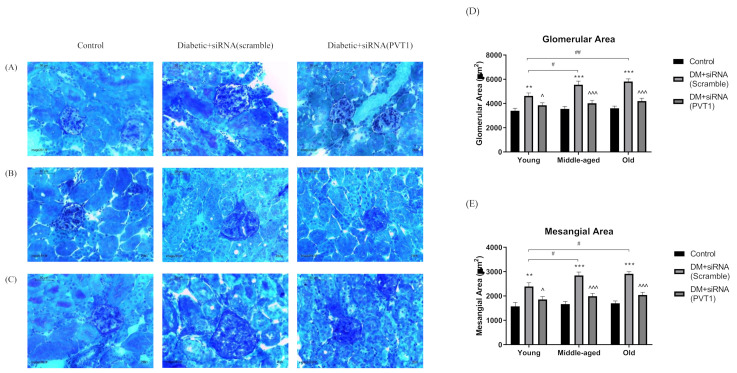
Microscopic images of kidney sections of siRNA-treated diabetic and control mice of different age groups: (**A**) young, (**B**) middle-aged and (**C**) old. Diabetic mice were treated with either scramble-siRNA (DM + siRNA (scramble)) or PVT1-siRNA (DM + siRNA (PVT1)), whereas control mice were normal mice without siRNA injection (Control). The kidney sections were stained with periodic acid Schiff (PAS) followed by Mayer’s hematoxylin and viewed under 40X. (**D**) Glomerular area and (**E**) mesangial area were semi-quantified using Image-J software. Data were presented as means ± SEM, n = 6. A *p*-value < 0.05 was considered statistically significant using one-way ANOVA with Tukey post-test (* *p* < 0.05, ** *p* < 0.01, *** *p* < 0.001 vs. age-matched control; ^ *p* < 0.05, ^^^ *p* < 0.001 vs. age-matched DM + siRNA (scramble); ^#^ *p* < 0.05, ^##^ *p* < 0.01 vs. young DM + siRNA (scramble).

The size and PAS-positive area of glomeruli were larger in diabetic mice with scramble-siRNA than in the age-matched controls or the diabetic mice with PVT1-siRNA (Figure 2A–C). The glomerular and mesangial areas were significantly greater in diabetic mice with scramble-siRNA in young (*p* < 0.01), middle-aged (*p* < 0.001) and old (*p* < 0.001) mice when compared with the age-matched controls, while moderate increase was observed in diabetic mice with PVT1-siRNA (Figure 2D,E). Compared with diabetic mice with scramble-siRNA, the glomerular and mesangial areas were markedly decreased in diabetic mice with PVT1-siRNA in young (*p* < 0.05), middle-aged (*p* < 0.001) and old (*p* < 0.001) groups. Both glomerular and mesangial areas were increased with advancing age in diabetic mice with scramble-siRNA.

### 3.6. Effect of Silencing PVT1 on ECM Accumulation

**Figure 3:**
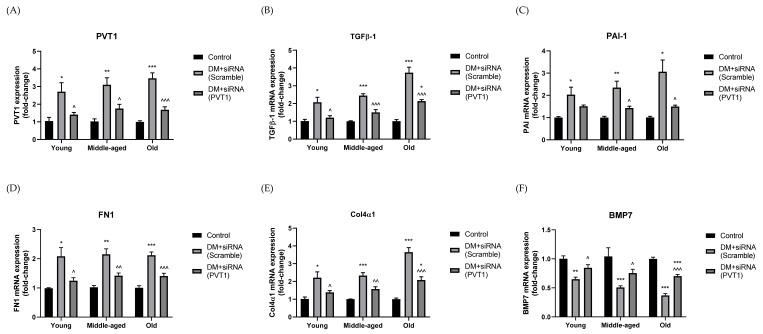
Gene expressions of (**A**) PVT1, (**B**) TGF-β1, (**C**) PAI-1, (**D**) FN1, (**E**) COL4A1, and (**F**) BMP7 of siRNA-treated diabetic and control mice of different age groups. Diabetic mice were treated with either scramble-siRNA (DM + siRNA (scramble)) or PVT1-siRNA (DM + siRNA (PVT1)), whereas control mice were normal mice without siRNA injection (Control). Data were presented as means ± SEM, n = 6. A *p*-value < 0.05 was considered statistically significant using one-way ANOVA with Tukey post-test (* *p* < 0.05, ** *p* < 0.01, *** *p* < 0.001 vs. age-matched control; ^ *p* < 0.05, ^^ *p* < 0.01, ^^^ *p* < 0.001 vs. age-matched DM + siRNA (scramble). PVT1 (Plasmacytoma variant translocation 1); TGF-β1 (Transforming growth factor beta 1); PAI-1 (Plasminogen activator inhibitor-1); FN1 (Fibronectin 1); COL4A1 (Collagen type IV alpha 1); BMP7 (Bone morphogenetic protein 7).

Gene expressions of PVT1 (Figure 3A), TGF-β1 (Figure 3B), PAI-1 (Figure 3C), FN1 (Figure 3D), and COL4A1 (Figure 3E) were significantly increased in diabetic mice with scramble-siRNA when compared with the age-matched controls, while that of BMP7 (Figure 3F) was markedly decreased. The changes in expressions became more obvious over time. With PVT1-siRNA treatment, expression of PVT1 was significantly decreased in young (by 48%, *p* < 0.05), middle-aged (by 43%, *p* < 0.05), and old (by 51%, *p* < 0.001) mice when compared with diabetic mice with scramble-siRNA. Likewise, expressions of TGF-β1, PAI-1, FN1 and COL4A1 were significantly downregulated when compared with diabetic mice with scramble-siRNA. Diabetic mice had significantly higher BMP7 expression after PVT1-siRNA treatment than that of scramble-siRNA treatment in all age groups. Expressions of PVT1, TGF-β1, PAI-1, FN1, COL4A1 and BMP7 were not statistically different between diabetic mice with PVT1-siRNA and the age-matched controls, except for TGF-β1, COL4A1 and BMP7 in the old age group.

## 4. Discussion

**Figure 4:**
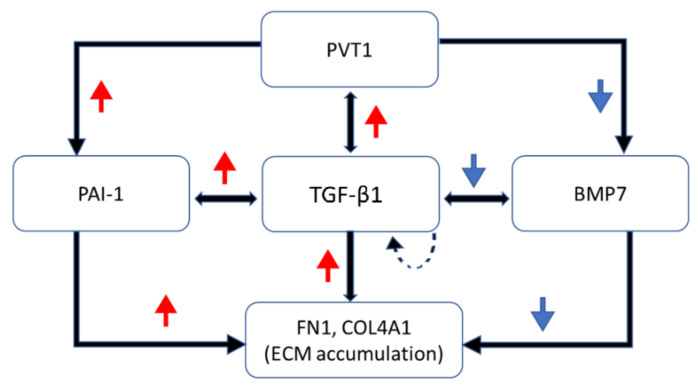
Potential role of PVT1 in ECM accumulation. Diabetes induces persistent hyperglycaemia in murine models. Hyperglycaemic or diabetic conditions induce an upregulation of PVT1 that results in the upregulation of ECM regulators (PAI-1 and TGF-β1) and ECM components (FN1 and COL4A1) while downregulating BMP7 in the glomeruli. PAI-1 is the main inhibitor of ECM degradation. TGF-β1 promotes ECM accumulation by upregulating PAI-1, FN1 and COL4A1. TGF-β1 also inhibits the anti-fibrotic actions of BMP7. PAI-1 and TGF-β1 regulate each other to simultaneously sustain the excessive ECM accumulation. In addition, the autocrine action of TGF-β1 to stimulate its own production further promotes its effects on ECM accumulation. PVT1 (Plasmacytoma variant translocation 1); TGF-β1 (Transforming growth factor beta 1); PAI-1 (Plasminogen activator inhibitor-1); FN1 (Fibronectin 1); COL4A1 (Collagen type IV alpha 1); BMP7 (Bone morphogenetic protein 7); red upward arrow represents a stimulatory response, blue downward arrow represents an inhibitory response.

For decades the focus of studies of the gene regulatory system has been mainly on the protein encoding genes, whereas genomic analyses have indicated that more than 90% of the mammalian genome is transcribed as non-coding RNAs [24]. Emerging evidence has shown that lncRNAs are not transcriptional noises but serve important functions in regulation of gene expression during normal development and disease progression [25]. Although PVT1 has been widely studied in a variety of cancer research, its role in DN is poorly understood. In disease status, PVT1 are overexpressed in human and animals with DN [16,17]. Likewise, our study has shown that PVT1 expression is significantly increased in mice with DN and its expression increases with the progression of DN over time. Expression of ECM regulators (TGF-β1 and PAI-1) and components (FN1 and COL4A1) are upregulated along with PVT1. TGF-β1 and PAI-1 are the main mediators of ECM accumulation, which is a pivot event of DN [26]. The expressed ECM regulators regulate each other while simultaneously sustaining the fibrotic response [27]. The autocrine action of TGF-β1 to stimulate its own production further promotes its effects on ECM accumulation [28]. Furthermore, TGF-β1 counteracts the anti-fibrotic actions of BMP7 (such as reducing FN1, COL4A1 and PAI-1) [29]. Silencing of PVT1 suppressed the diabetic-induced upregulation of ECM components (FN1 and COL4A1) and regulators (PAI-1 and TGF-β1) while restoring BMP7 expression. The potential role of PVT1 in ECM accumulation is suggested in Figure 4. In addition, silencing of PVT1 suppresses kidney hypertrophy, proteinuria (elevation of UAE, UACR, UPE and UPCR), kidney function decline (reduced creatinine clearance and elevated serum creatinine) and histological changes (increased glomerular and mesangial areas) in diabetic mice.

The creatinine clearance in young diabetic mice was slightly reduced when compared with its control. This could be explained by the compensatory changes in surviving nephrons after the nephron loss in kidney injury, which are commonly observed in the clinical practice. The GFR was decreased by only 20–30% when half of the functioning nephrons were injured [30]. The functional compensation is likely to resume a normal GFR until its capacity is exceeded. Therefore, the effect of PVT1 inhibition on creatinine clearance in young diabetic mice was not as obvious as in older ones and other hallmarks of DN developed gradually as the diabetic mice aged.

A recent study has shown that PVT1 inhibition suppressed renal fibrosis through inactivation of TGF-β signalling in UUO-induced murine model [31]. Although the UUO-induced model mimicking human chronic obstructive nephropathy is different from the diabetic model, the resulting fibrotic response is a common pathway for most forms of progressive renal disease, including DN. The positive association between PVT1 and TGF-β1 expression shown in the present study has suggested that silencing of PVT1 suppresses TGF-β1-mediated renal fibrosis in DN. This is further supported by the significant decline in serum TGF-β1 levels in DN models with PVT1-siRNA treatment, against those without treatment, in all age groups. This might suggest a renoprotection where the circulating TGF-β1 level is considered as a good predictor of DN progression in diabetic patients [32].

The present study demonstrates that silencing of PVT1 did not significantly reduce hyperglycaemia in diabetic mice but did suppress the decline in insulin levels. PVT1 was suggested to regulate insulin sensitivity, lipid metabolism and adipogenesis in different models [33,34]. Hyperglycaemia is one of risk factors for DN development. However, many individuals with relatively modest hyperglycaemia develop DN, whereas some with prolonged hyperglycaemia never develop DN [35]. Clinical trials have shown that antihyperglycemic and antihypertensive/antiproteinuric control are useful in the prevention of DN progression in diabetic patients [36]. Although the effects of PVT1 on hyperglycaemia and hypertension are not clear, our study has showed that proteinuria and kidney function decline were prevented by PVT1 inhibition. Furthermore, elevated insulin levels by PVT1 inhibition might contribute to reducing the ECM accumulation as insulin was reported to decrease synthesis of FN and ECM assembly [37]. In summary, silencing of PVT1 is suggested to provide renoprotection against DN through suppressing ECM accumulation, TGF-β1 elevation and insulin decline.

There were some limitations in this study. Based on the recommendation of Animal Models of Diabetic Complications Consortium (AMDCC), an ideal DN model should fulfil three criteria: (1) more than 50% decline in GFR over the lifetime of the animal; (2) more than 10-times higher albuminuria compared with the strain, gender and age-matched controls; (3) pathological features including advanced mesangial matrix expansion, GBM thickening, arteriolar hyalinosis and tubulointerstitial fibrosis [38]. However, no animal model in the literature has met all criteria so far [39]. The murine DN model adopted in this study partly satisfied the clinical features in that the GFR evaluated as creatinine clearance was approaching 50% decline at 24 weeks old and the high-grade albuminuria of 2.0–3.3-fold increase compared with the age-matched controls. Arteriolar hyalinosis, nodular glomerulosclerosis, or Kimmelstiel-Wilson lesion is found in humans with advanced DN but are generally absent in mice [38,40]. Secondly, the renoprotection of PVT1 silencing on female mice was likely yet unreported, as only male C57BL/6 mice were used, which are more susceptible to kidney damage [41]. Despite this, the murine DN models established by the combination treatment of STZ and high fat diet exhibited progressive renal insufficiency, albuminuria and mesangial expansion over time, which are clear indications of DN progression from early to advanced stages. Thirdly, the relative renoprotection of PVT1 silencing with respective to that of positive reference or anti-diabetic drug treatment was undetermined, as the latter was not included. It is worthwhile to note that anti-diabetic drugs treating advanced DN may be questionable as their glucose-modulating effects lessen with declining GFR [42]. The histological collagen deposition should be evaluated in addition to mesangial and glomerular areas [43]. Lastly, PVT1 may exert its effects on DN progression through other mechanisms besides ECM accumulation, such as podocyte apoptosis [16], inflammation and oxidative stress [44], and haemodynamic factors [45]. Further investigations are needed to delineate the complex network of PVT1 and its interactions with other biological molecules.

RNAi-based therapeutics are emerging treatment strategies in cancers, infectious diseases and single-gene disorders [46]. Currently, many clinical trials using various siRNAs or shRNAs have been conducted [47]. The first clinical trial using systemic administration of a siRNA for acute kidney injury has proceeded to subsequent phase trials with favorable safety data [48]. Our study provided a relatively long-term RNAi-based treatment to the DN models when compared to some previous animal studies. The importance of lncRNA studies is noted even with the current limited knowledge. Although the potential for siRNA therapy is recognized, further scientific research is needed to investigate the development of DN-related RNAi technology and translational applications in clinical settings.

## 5. Conclusions

A murine DN model was established by a combination of low-dose STZ injection and high fat diet, which, although exhibiting different extents of hyperglycaemia and albuminuria, reduced creatinine clearance, kidney hypertrophy, and increased glomerular and mesangial areas. Silencing of PVT1 ameliorates DN in terms of kidney function and histology in diabetic mice of young, middle and old ages. The renoprotection is suggested to be attributable to the reduced glomerular COL4A1 and FN1 expression through suppressing of TGF-β1 and PAI-1 while preserving of BMP7 expression, as well as reduced serum TGF-β1 and prevention of insulin decline. It is therefore suggested that PVT1 affects ECM accumulation and is a potential target for the treatment of DN. Further study is needed to characterize the complex molecular mechanism of PVT1 in the context of DN progression. The present study also demonstrates the application of RNAi-based therapy to delay DN progression in murine DN models, which suggests the potential for further investigation and development.

## Figures and Tables

**Table 1:** Physical and biochemical characteristics of diabetic and control mice of different age groups. The diabetic group and normal control groups were represented by the HFD-STZ-treated mice and the LFD-vehicle-treated mice, respectively. Data were presented as means ± SEM, n = 6. A *p*-value < 0.05 was considered statistically significant using unpaired Student’s *t*-test (* *p* < 0.05, ** *p* < 0.01, *** *p* < 0.001 vs. age-matched control) or one-way ANOVA with Tukey post-test (^#^ *p* < 0.05, ^###^ *p* < 0.001 vs. young diabetic). UAE (Urine Albumin Excretion); UACR (Urine Albumin to Creatinine Ratio); UPE (Urine Protein Excretion); UPCR (Urine Protein to Creatinine Ratio).

	Young (9 Weeks)		Middle-Aged (16 Weeks)		Old (24 Weeks)	
	Control	Diabetic	Control	Diabetic	Control	Diabetic
Body weight (g)	26.090 ± 0.580	28.030 ± 0.560 *	28.530 ± 0.460	30.410 ± 0.600 *	31.850 ± 0.900	34.120 ± 1.530
Body weight change (%)	32.990 ± 3.270	46.200 ± 3.830 *	48.590 ± 4.560	66.360 ± 5.760 *	67.850 ± 4.900	80.040 ± 10.470
Blood glucose (mmol/l)	10.83 ± 0.41	25.61 ± 0.42 ***	12.44 ± 0.41	28.37 ± 0.90 ***	11.58 ± 0.26	27.07 ± 1.79 ***
Kidney weight (g)	0.228 ± 0.011	0.268 ± 0.022	0.234 ± 0.009	0.285 ± 0.018 *	0.238 ± 0.008	0.275 ± 0.015 *
Kidney to body weight ratio	0.009 ± 0.000	0.001 ± 0.000	0.008 ± 0.000	0.009 ± 0.000 *	0.008 ± 0.000	0.008 ± 0.000
Serum creatinine (mg/dl)	0.53 ± 0.03	0.81 ± 0.09 *	0.57 ± 0.04	0.93 ± 0.12 *	0.58 ± 0.04	0.95 ± 0.14 *
Creatinine clearance (ml/min)	0.022 ± 0.002	0.020 ± 0.003	0.0220 ± 0.002	0.014 ± 0.002 *	0.021 ± 0.003	0.012 ± 0.001 *^,^^#^
UAE (μg)	9.680 ± 1.200	19.940 ± 2.670 **	11.100 ± 2.670	33.230 ± 2.940 ***^,^^#^	15.360 ± 2.470	50.550 ± 4.910 ***^,^^###^
UACR	0.064 ± 0.010	0.098 ± 0.011 *	0.073 ± 0.0047	0.152 ± 0.011 ***^,^^#^	0.088 ± 0.005	0.219 ± 0.018 ***^,^^###^
UPE (mg)	0.527 ± 0.055	0.799 ± 0.133	0.520 ± 0.123	0.968 ± 0.125 *	0.530 ± 0.069	1.074 ± 0.134 *
UPCR	3.093 ± 0.090	4.072 ± 0.421 *	3.051 ± 0.343	4.288 ± 0.325 *	2.911 ± 0.308	4.420 ± 0.306 **

**Table 2:** Physical and biochemical characteristics of siRNA-treated diabetic and control mice of different age groups. Diabetic mice were treated with either scramble-siRNA (DM + siRNA (scramble)) or PVT1-siRNA (DM + siRNA (PVT1)), whereas control mice were normal mice without siRNA injection (Control). Data were presented as means ± SEM, n = 6. A *p*-value < 0.05 was considered statistically significant using one-way ANOVA with Tukey post-test (* *p* < 0.05, ** *p* < 0.01, *** *p* < 0.001 vs. age-matched control; ^ *p* < 0.05, ^^ *p* < 0.01, ^^^ *p* < 0.001 vs. age-matched DM + siRNA (scramble); ^#^ *p* < 0.05, ^##^ *p* < 0.01, ^###^ *p* < 0.001 vs. young DM + siRNA (scramble); ^@^ *p* < 0.05, ^@@^ *p* < 0.01 vs. young DM + siRNA (PVT1)). UAE (Urine Albumin Excretion); UACR (Urine Albumin to Creatinine Ratio); UPE (Urine Protein Excretion); UPCR (Urine Protein to Creatinine Ratio).

	Young (9 Weeks)			Middle-Aged (16 Weeks)			Old (24 Weeks)		
	Control	DM + siRNA (Scramble)	DM + siRNA (PVT1)	Control	DM + siRNA (Scramble)	DM + siRNA (PVT1)	Control	DM + siRNA (Scramble)	DM + siRNA (PVT1)
Body weight (g)	22.930 ± 0.230	26.600 ± 0.860 *	26.230 ± 0.470 *	27.630 ± 0.760	30.300 ± 0.460 *	28.000 ± 0.530 ^	28.370 ± 0.410	29.820 ± 0.500	30.170 ± 0.310
Body weight change (%)	25.350 ± 1.310	40.490 ± 1.400 ***	34.580 ± 1.210 **^,^ ^	47.460 ± 3.600	70.670 ± 2.620 **	50.590 ± 3.810 ^^	92.390 ± 4.180	101.730 ± 9.520	96.130 ± 2.500
Blood glucose (mmol/l)	12.37 ± 0.67	25.42 ± 0.77 ***	23.47 ± 0.42 ***	12.10 ± 0.35	30.27 ± 0.35 ***	28.82 ± 0.36 ***^,^ ^	11.90 ± 0.44	28.38 ± 0.52 ***	26.74 ± 0.50 ***
Kidney weight (g)	0.252 ± 0.002	0.258 ± 0.008	0.240 ± 0.005	0.236 ± 0.012	0.266 ± 0.012	0.250 ± 0.009	0.240 ± 0.011	0.269 ± 0.005	0.262 ± 0.004
Kidney to body weight ratio	0.010 ± 0.000	0.010 ± 0.001	0.009 ± 0.000	0.009 ± 0.000	0.011 ± 0.000 **	0.0090 ± 0.000 ^^	0.009 ± 0.000	0.011 ± 0.000 **	0.008 ± 0.000 ^^^
Serum TGF-β1 (ng/ml)	72.95 ± 2.58	90.69 ± 3.58 **	76.31 ± 2.17 ^	78.61 ± 4.38	96.70 ± 2.72 *	83.34 ± 2.76 ^	78.61 ± 4.38	96.70 ± 2.72 **^,^^#^	83.34 ± 2.76 ^^,^^@^
Serum insulin (ng/ml)	1.386 ± 0.202	0.241 ± 0.140 **	0.750 ± 0.193	1.396 ± 0.249	0.299 ± 0.086 **	0.626 ± 0.204 *	1.148 ± 0.357	0.120 ± 0.026 **	0.274 ± 0.121 *
Serum creatinine (mg/dl)	0.667 ± 0.037	1.015 ± 0.001 ***	0.836 ± 0.031 ^	0.702 ± 0.062	1.318 ± 0.102 ***	0.991 ± 0.063 ^	0.700 ± 0.094	1.168 ± 0.069 **	0.936 ± 0.087
Creatinine clearance (ml/min)	0.032 ± 0.002	0.028 ± 0.002	0.031 ± 0.004	0.032 ± 0.002	0.020 ± 0.002 *^,^^#^	0.028 ± 0.003	0.031 ± 0.004	0.016 ± 0.001 **^,^^##^	0.025 ± 0.002 ^
UAE (μg)	11.760 ± 0.290	25.923 ± 1.043 ***	16.253 ± 1.644 ^^^	13.071 ± 0.397	38.817 ± 3.094 ***^,^^#^	22.272 ± 2.911 ^^	14.518 ± 0.481	44.337 ± 4.255 ***^,^^##^	24.632 ± 2.278 ^^
UACR	0.067 ± 0.009	0.135 ± 0.015 **	0.069 ± 0.008 ^^	0.081 ± 0.006	0.162 ± 0.014 **^,^^#^	0.110 ± 0.017 ^	0.094 ± 0.016	0.233 ± 0.021 ***^,^^##^	0.132 ± 0.011 ^^^,^^@@^
UPE (mg)	0.808 ± 0.051	0.989 ± 0.068	0.834 ± 0.041	0.913 ± 0.090	1.679 ± 0.084 **^,^^#^	1.015 ± 0.150 ^^	1.046 ± 0.089	2.067 ± 0.198 **^,^^###^	1.433 ± 0.175 ^^,^^@^
UPCR	2.845 ± 0.184	3.331 ± 0.165	2.997 ± 0.219	2.833 ± 0.160	3.569 ± 0.217 *	3.138 ± 0.099	2.873 ± 0.249	3.976 ± 0.315 *	3.127 ± 0.177

## Data Availability

The datasets used and/or analyzed during the current study are available from the corresponding author on reasonable request.
